# Genetically Modified Organisms and Visceral Leishmaniasis

**DOI:** 10.3389/fimmu.2014.00213

**Published:** 2014-05-14

**Authors:** Rudra Chhajer, Nahid Ali

**Affiliations:** ^1^Infectious Diseases and Immunology Division, Council of Scientific and Industrial Research-Indian Institute of Chemical Biology, Kolkata, India

**Keywords:** vaccines, immunology, *Leishmania*, genetically modified parasites, visceral leishmaniasis

## Abstract

Vaccination is the most effective method of preventing infectious diseases. Since the eradication of small pox in 1976, many other potentially life compromising if not threatening diseases have been dealt with subsequently. This event was a major leap not only in the scientific world already burdened with many diseases but also in the mindset of the common man who became more receptive to novel treatment options. Among the many protozoan diseases, the leishmaniases have emerged as one of the largest parasite killers of the world, second only to malaria. There are three types of leishmaniasis namely cutaneous (CL), mucocutaneous (ML), and visceral (VL), caused by a group of more than 20 species of *Leishmania* parasites. Visceral leishmaniasis, also known as kala-azar is the most severe form and almost fatal if untreated. Since the first attempts at leishmanization, we have killed parasite vaccines, subunit protein, or DNA vaccines, and now we have live recombinant carrier vaccines and live attenuated parasite vaccines under various stages of development. Although some research has shown promising results, many more potential genes need to be evaluated as live attenuated vaccine candidates. This mini-review attempts to summarize the success and failures of genetically modified organisms used in vaccination against some of major parasitic diseases for their application in leishmaniasis.

## Introduction

**Figure 1:**
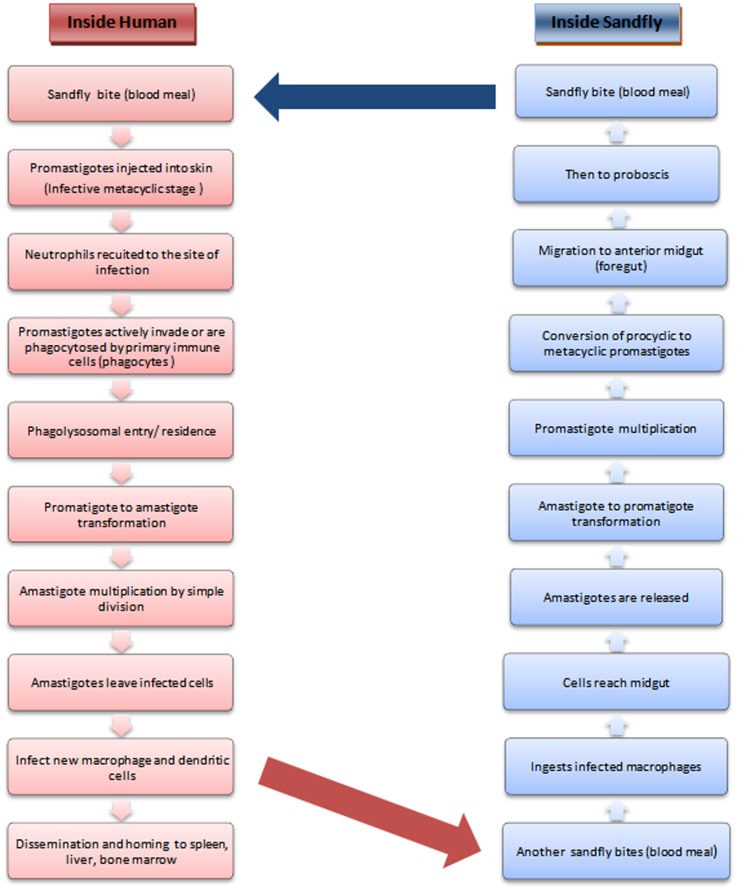
**Life cycle of *Leishmania***.

The leishmaniases comprise a group of largely neglected tropical diseases, transmitted during the blood meal of the phlebotomine sandfly (Figure 1). The disease outcome ranges from the mild cutaneous, more severe mucocutaneous to the almost fatal visceral leishmaniasis (followed by PKDL in a small proportion of VL patients) depending upon the transmitted species of *Leishmania* parasite. With more than 90% of the VL patients concentrated in south-east Asia and Africa, the statistics indicate that almost 200 million people are at risk worldwide, which is only a rough estimate, as a major population remains asymptomatic and hence unrecognized (1). VL ranks fourth in morbidity among all tropical diseases with an annual incidence of 2.5/1000 persons (2) and is second only to malaria in terms of mortality (3).

Despite abundant research in recent years, the available treatment options are far from satisfactory. The drugs are associated with toxicity, high cost, and/or resistance. In this context, multi-drug combinatorial therapies have shown some promise (4). Prevention by vaccination is favored by the fact that healing from leishmaniasis is almost always associated with lifelong resistance to infection. A desirable vaccine would provide long term immunity; elicit a T-cell immune response that would be a balance of Th1 mediated immune activation against the pathogen and Th2 mediated suppression to avoid excess tissue damage, produce a strong memory and effector response upon subsequent challenge, be persistent, and highly immunogenic (3). However, the vaccine should not elicit an auto-immune response and be safe even in immune-compromised SCID mice and HIV patients (5).

Based on the general nature of the formulation, there are three types of anti-leishmanial vaccines (6). The first generation of vaccines is comprised of live, virulent parasites injected at hidden body parts so as to avoid lesion visibility (leishmanization) or of inactivated parasites achieved by heat, radiation, antibiotics, chemical mutagenesis, and selection for temperature sensitivity or long passages in-culture (7). The second generation includes crude whole cell lysates, purified fractions, or subunit vaccines composed of single or multiple recombinant or native antigens. The only approved vaccine for human trial is Leish111f, a multivalent vaccine, composed of a thiol-specific antioxidant, *Leishmania major* stress inducible protein 1, and *L. major* elongation initiation factor (8). The third generation of vaccines consists largely of DNA in the form of mammalian expression plasmids or viral vectors encoding virulence factors (9). Unfortunately, the efficacy of available DNA and protein subunit vaccine candidates are limited (10). Recent concepts introduce the use of sandfly salivary antigens, T-cell epitope based peptides, antigen pulsed DC’s, and genetically modified live attenuated parasites (11). In contrast, vaccination using live attenuated parasites mimics natural infection and overcomes most of these limitations (12). Additionally, their persistence and display of parasites entire antigenic repertoire alleviates the need for an adjuvant. The recent success of live attenuated vaccination (LAV) in malaria, the clear genetic profile, and safety from reversion of complete knock-outs further encourages this endeavor.

## Genetic Modification in *Leishmania*: Applications and Types

Due to advances in axenic parasite culture, transfection efficiency, availability of genetic manipulation vectors (for expression, recombination, or integration), and the plethora of sequence based information available (from databases, like GeneDB, LeishCyc, LeishBase, KEGG, TriTrypDB, and TDR Targets), the ease and scope of creating live attenuated parasites has increased tremendously (13). Such parasites can be used to elucidate novel drug targets as well as vaccine candidates based on whether the gene under study is essential for both the promastigote and amastigote stages of the parasite or, only the amastigote stage. In addition, genetically modified organisms can also be used in metabolic pathways studies, structure-function relationship investigates (14), screening of new drugs (15), host–parasite interaction, and post-infection analysis among others, to enhance our understanding of these lower eukaryotes. Considering the success of LAV strategies against many viral, bacterial, and protozoan diseases (although to different extents), these are now considered the gold standard for protection against intra-cellular pathogens (12).

Foreign or self genes can be introduced in either episomal or integrated form, for expression of particular proteins to study their effects on various aspects of the parasites life cycle. In the episomal form, the gene’s expression is under the control of the vector specific promoter, which can be inducible or not (for stage specific expression analysis). For integration, the genes are generally targeted downstream of the ribosomal RNA locus to study the effects of constitutive expression at all stages of the life cycle. In either case, the genes can be fused to fluorescent reporter genes for ease of monitoring their expression (15). In addition, there are methods to selectively knock-out particular regions of interest heterologously or homologously using gene specific targeting constructs (16–18). During deletion, the targeted region is replaced by an antibiotic selection marker. Its expression makes the modified cells resistant to that antibiotic, thereby facilitating selection. Multiple genes can be targeted simultaneously. This exchange is generally brought about by the double strand break repair model of homologous recombination (19) whose major role has been the maintenance of its multi-gene families, conferring a selective advantage to parasites stressed by antifolate drugs (by upregulation of resistance genes) (14, 16). Alternatively, the transcripts of the genes can also be simply knocked down by anti-sense RNA interference technique, thereby blocking translation. However, with a few exceptions most leishmanial species lack the RNAi machinery (20).

## Success of Live Attenuated Vaccination in Other Diseases

Herein, we will discuss LAV strategies in various mosquito borne, viral, protozoal, and bacterial diseases. Malaria, which exerts significant mortality, morbidity, and economic burden, is spread by intra-cellular parasitic apicomplexans of the genus *Plasmodium*. Like *Leishmania, Plasmodium* has multiple hosts and forms and rapid amplification is key to its survival and spread. Their pathogenic liver and transmission stages have been the most often chosen targets for attenuation because compared to the blood stages, they are low in numbers and exhibit limited antigenic variation, making it less probable that a vaccine will fail against heterologous parasite strains. The search for a live attenuated malaria vaccine provided some invaluable insights that can be applied to leishmanial as well as other infectious diseases. The failure of the inactivated sporozoites, and success of γ-irradiated ones, demonstrated the requirement of live and host cell invasive parasites to confer protection (21–23). The ability of the *UIS3*^−/−^ sporozoites to confer protection against sporozoite re-infection but not blood stage transfusion, demonstrates stage specific immunity, herein, liver stage. Hence, not all stages of a parasites cycle may be equally useful for LAV approaches (24). The deletion of liver stage specific fatty acid synthesis pathway genes, however, had no effect on replication and gametogenesis, indicating that only essential metabolic pathways should be targeted for attenuation. Furthermore, multiple deletions sometimes may be more effective, as combined *p26/p52* knock-out provided better protection than either of the single knock-outs in both chimeric mouse harboring human hepatocytes as well as both low/high dose human trials (22, 25, 26). These mutants exhibited complete growth arrest during the liver stages. However, their pre-erythrocytic stages were unhampered, thereby not hindering the possibility of large-scale production. Similarly, for leishmania, an unaffected promastigote growth stage would be desirable for a strain to be used for vaccination.

Another virus that largely affects the cloven hoofed animals worldwide is the foot and mouth disease virus (FMDV). Control by limiting animal movements and herd destruction has been mostly practiced due to insufficient protection by the available inactivated vaccine against all three FMDV variants. Recently, however, a reverse genetics approach has yielded a novel vaccine candidate by substitutions in a few amino-acids showing remarkable protection. These mutants too had normal growth properties as desirable for large-scale vaccine production (27).

One of the most successful and oldest examples of live attenuated vaccines is the 17D strain of yellow fever virus. It has also served as a model for vaccination strategies against dengue, a viral disease caused by transmission of one of its four serotypes 1–4 by the *Aedes* mosquito. Sanofi Pasteur’s ChimeriVax Dengue tetravalent vaccine (CVD1–4) is the most advanced product so far and a chimera in the truest sense utilizing the licensed YFV 17D vaccine as backbone, each expressing the *prM* and *E* genes of one of the four DENV serotypes. An effective dengue vaccine should consist of a tetravalent formulation, with components representing each serotype (28). A “stem-loop” genomic region implicated in its pathogenicity has been deleted to create the rDEN(1,2,4)Δ30 strains that impart adequate protection. However, the rDEN3Δ30 was not protective, indicating differences among strains. Hence, a novel chimerization led to a creation of rDEN3/4Δ30(ME) – a recombinant virus backbone of serotype 4 with Δ30 deletion, containing the *ME* region of a naturally attenuated serotype 3 strain, having manifold lower replication and transmission. This is a perfect example of successful extrapolation from sabin polio virus whose second component was also a naturally attenuated polio strain (29).

The MMR vaccine against measles, mumps, and rubella given to expecting mothers is another successful example of a multivalent vaccine that reduces the number of doses and avoids unnecessary delays and problems of spacing live attenuated vaccines (30). With pandemic capacity (31), the influenza vaccine, has been a huge challenge with its constantly varying epitopes resulting in antigenically drifted strains (32). In such cases, focusing on the most constant regions is the best strategy. However, till a strain specific vaccine is available, reasonable protection can be offered by a recombinant adenoviral vector expressing antigens from H5, H7, and H9 avian influenza virus strains (33). The success of multivalent, dengue, influenza, and MMR vaccines offers the idea for such a vaccine against CL, ML, and VL too.

Among bacteria, *Streptococcus suis*, that causes swine flu is a global health hazard to the swine industry, associated with septic shock, pneumonia, meningitis, and arthritis. The current vaccine against it is a Sly gene deletion attenuated strain undergoing some refinement by association with other surface antigens and adjuvants (34). The Bacillus Calmette Guerin vaccine for tuberculosis is created by long *in vitro* passaging of the intra-cellular bacteria *Mycobacterium tuberculosis*. The gradual loss of the RD loci has been reported as the major cause for this attenuation. Hence, attempts at manually creating these deletions are on. Recombinant BCG vaccines co-expressing other antigens from pathogens are also in clinical trials (35, 36). For cholera too, many endogenously produced live attenuated vaccines (Peru15 and Bengal15) are available as a traveler’s vaccine in different countries (37–39).

## Elucidation of Novel Vaccine Candidates and Drug Targets: Attempts Made in *Leishmania*

In contrast to leishmanial species causing CL, research on genetic modification in VL has been limited. However, recent years have seen a significant improvement in this scenario (Table 1). Though mostly focused at elucidating metabolic pathways, cellular processes, and host–parasite interactions; it has simultaneously led to the discovery of novel drug targets and vaccine candidates. The major pathways targeted were those that are unique to the parasite’s life cycle or metabolism, components sufficiently different from the homolog in hosts. Today, bio-informatic databases, proteomic screens (40), and reverse vaccinology, aid in the identification of novel vaccine candidates based on their expression stage, abundance, sub-cellular localization, sequence conservation in leishmanial species, non-homology to their human counterparts, trans-membrane helix predictions, and T-cell epitopic regions (12). Using the same genetically modified strain, research collaborations between labs working on different aspects of leishmaniasis can greatly speed up and enhance this search. Some of the most important pathways and their components, that have surfaced, are briefly discussed below.

**Table 1:** **Genetic deletions that led to the discovery of novel drug or vaccine candidates in VL causing organisms**.

Organism	Target gene	Animal model	Immune response	Persistence	Inference		Reference
					Drug	LAV	
*L. mexicana*	Arginase	NA	NA	NA	+	UC	(41–43)
*L. major*							
*L. donovani*	Ornithine decarboxylase	BALB/c mice	Reduced virulence *in vitro* and *in vivo*	NA	+	+	(44–46)
*L. donovani*	Spermidine synthase	BALB/c mice	Decreased organ parasite burden	4 weeks	+	UC	(47)
*L. donovani*	*S*-adenosylmethionine decarboxylase	NA	NA	NA	+	UC	(48)
*L. donovani*	Trypanothione reductase	NA	Reduced virulence *in vitro*	NA	+	UC	(49–52)
*L. donovani*	Trypanothione synthetase	NA	NA	NA	+	UC	(42, 53)
*L. donovani*	Hypoxanthine–guanine phosphoribosyl transferase	NA	No effect on virulence *in vitro* and *in vivo*	NA	X	X	(54)
*L. donovani*	Adenine phosphoribosyl transferase	NA	No effect on virulence *in vitro* and *in vivo*	NA	X	X	(54, 55)
*L. donovani*	Xanthine phosphoribosyl transferase	NA	No effect on virulence *in vitro* and *in vivo*	NA	+	UC	(54, 56)
*L. donovani*	Inosine monophosphate dehydrogenase	BALB/c mice	No effect on virulence *in vivo*	NA	X	X	(57)
*L. donovani*	Adenine aminohydrolase	BALB/c mice	No significant effect on parasitemia *in vitro* or in organ parasite burden	NA	+	UC	(58)
*L. donovani*	Hypoxanthine–guanine phosphoribosyl transferase/xanthine phosphoribosyl transferase	NA	Highly reduced virulence *in vitro*	NA	–	+	(59)
*L. donovani*	Adenine aminohydrolase/hypoxanthine–guanine phosphoribosyl transferase/xanthine phosphoribosyl transferase	BALB/c mice	Avirulent *in vitro* and *in vivo*	4 weeks	–	+	(58)
*L. donovani*	Adenylosuccinate synthetase	BALB/c mice	Reduced virulence *in vitro* but not *in vivo*	NA	X	X	(60)
*L. donovani*	Adenylosuccinate lyase	BALB/c mice	Reduced virulence *in vitro* and *in vivo*	NA	+	UC	(60)
*L. donovani*	Uridine monophosphate synthase	NA	NA	NA	+	UC	(61)
*L. donovani*	Uracil phosphoribosyl transferase	BALB/c mice	No effect on virulence *in vitro* or *in vivo*	NA	+	UC	(62, 63)
*L. donovani*	Carbamoyl phosphate synthetase	BALB/c mice	Reduced virulence *in vitro* and decreased parasite burden	NA	+	UC	(62)
*L. donovani*	Uracil phosphoribosyl transferase/carbamoyl phosphate synthetase	BALB/c mice	Reduced virulence *in vivo*	4 weeks	–	+	(62)
*L. donovani*	Biopterin transporter 1	BALB/c mice	Reduced virulence *in vivo*. Protective against challenge infection. Increased IFN-γ production upon splenocyte stimulation	3 months	UC	+	(64)
*L. donovani*	Centrin	BALB/c mice, SCID mice, golden Syrian hamsters	Long term protection against challenge infection-early clearance. Protective Th1-type immune response. Increase of single and multiple cytokine (IFN-γ, IL-2, and TNFα) producing cells, IFN-γ/IL-10 ratio, IgG2a immunoglobulins and NO production. Reduced organ parasite burden. Cross-protective against *L. braziliensis* challenge	10 weeks	UC	+	(65, 66)
*L. donovani*	P27, a cytochrome *c* oxidase component	BALB/c mice	Reduced virulence *in vivo*. NO generation, Ag-specific multifunctional CD4 and CD8 T-cells, enhanced secretion of pro-inflammatory cytokines IFN-γ, TNF-α, IL-12, and anti-inflammatory cytokines IL-10, IL-4, and IL-13	20 weeks	UC	+	(67)
*L. donovani*	Ubiquitin fold modifier-1	NA	Reduced virulence in human macrophages	NA	+	+	(68)
*L. donovani*	Golgi GDP mannose transporter	BALB/c mice	Reduced virulence *in vitro* and *in vivo*	Long term	+	+	(69)
*L. donovani*	Amastigote specific expression protein-2	BALB/c mice	Decreased virulence *in vitro* and *in vivo*	NA	+	X	(70)
*L. donovani*	Cathepsin b cysteine protease	NA	Decreased virulence in U937 macrophage cells	NA	+	UC	(71)
*L. donovani*	Oligopeptidase b serine protease	BALB/c mice	Decreased virulence in the murine footpad infection model. Massive upregulation in gene-transcription	NA	+	UC	(72)
*L. donovani*	Subtilisin protease	BALB/c mice, golden Syrian hamsters	Reduced virulence *in vivo*	NA	+	UC	(73)
*L. donovani*	Myosin	NA	NA	NA	+	UC	(74)
*L. donovani*	70 kDa subunit of outer dynein arm docking complex	NA	Increased virulence *in vitro*	NA	X	X	(75)
*L. donovani*	Actin	NA	Reduced survival *in vitro* mice peritoneal macrophage cells	NA	+	UC	(76)
*L. donovani*	ADP-ribosylation factor like protein-3A	NA	NA	NA	+	UC	(77)
*L. infantum*	Heat shock protein 70 type II	*L. major* model of infection in BALB/c mice, SCID mice, golden Syrian hamster	Increased NO production and protection by type 1 immune response in BALB/c mice	NA	UC	+	(78)
*L. donovani*	Small glutamine rich tetra trichopeptide	NA	NA	NA	+	UC	(79)
*L. donovani*	Casein kinase 1 isoform 4	NA	Increased virulence *in vitro* mice peritoneal macrophage cells	NA	+	UC	(80)
*L. donovani*	Glyoxalase I	NA	NA	NA	+	UC	(81)
*L. donovani*	cyp5122A1, a cytochrome P450	Golden Syrian hamsters	Decreased virulence *in vitro* and *in vivo*	NA	+	UC	(82)

**Color codes**	**Role**	**Symbols/short forms**	**Interpretation**				

Purple	Polyamine metabolism	NA	Not available				
Blue	Purine metabolism	+	Positive indication				
Gray	Pyrimidine metabolism	–	Not evaluated				
Green	Amastigote stage	UC	Uncertain				
Yellow	Protease	X	Negative indication				
Peach	Cytoskeletal involvement						
Pink	Chaperones						
White	Others						

### Polyamine metabolism

Polyamines are essential for proliferative processes and trypanothione synthesis. Their biosynthesis involves arginase, ornithine decarboxylase, *S*-adenosylmethionine decarboxylase, and spermidine synthase. In *Leishmania*, spermidine along with trypanothione reductase and trypanothione synthetase replace the antioxidant pathways of the host and are necessary for survival. Deletion of any of these enzymes implicates the essentiality of polyamine biosynthesis in both promastigotes and amastigotes, rendering them important drug targets.

### Nucleotide metabolism

Purines and pyrimidines are indispensable to all life. However, *Leishmania* are purine auxotrophs. Surprisingly, deletion of any of the purine salvages enzymes, namely hypoxanthine–guanine phosphoribosyl transferase (*Hgprt*), adenine phosphoribosyl transferase (*Aprt*), and xanthine phosphoribosyl transferase (*Xprt*); guanylate nucleotide synthesis enzyme namely inosine monophosphate dehydrogenase (IMPDH) or; adenine aminohydrolase (*Aah*) does not prove their essentiality for either salvage, virulence, or viability. However, multiple knock-out strains such as Δ*hgprt/*Δ*xprt* and Δ*aah/*Δ*hgprt/*Δ*xprt* are avirulent and hence potential vaccine candidates. However, the upregulation of *Xprt* in combined mutants implicate their therapeutic potential. Similarly, although both adenylosuccinate synthetase (*Adss*) and adenylosuucinate lyase (*Asl*) null mutants show diminished virulence, only the Δ*asl* null mutants are profoundly incapacitated in their ability to infect mice and essential for purine salvage by both life cycle stages.

In contrast to purines, *Leishmania* are prototrophic for pyrimidines. Nevertheless, they also possess some salvage enzymes. Deletion of the uridine monophosphate synthase (*Umps*), a bifunctional enzyme for UMP biosynthesis established this enzyme as essential for pyrimidine biosynthesis. Additionally, although single deletions of either uracil phosphoribosyl transferase (*Uprt*) or carbamoyl phosphate synthetase (*Cprt*) did not affect parasite growth, their combined deletion mutants were completely attenuated exhibiting reduced survivability, hence potential live vaccine candidates.

### Amastigote stage specific proteins

Amastigote stage specific genes are considered good targets for attenuation. Vaccination with null mutants of the biopterin transporter 1 (*Bt1*) gene, involved in biopterin transport; centrin (*Cen*), involved in the cell division cycle; *p27*, a cytochrome *c* oxidase complex component; *Lpg-2* (Golgi GDP mannose transporter), involved in phosphoglycan synthesis, which is essential for host–parasite interactions or ubiquitin fold modifier-1 (*Ufm-1*) gene involved in fatty acid metabolism produced a strong protective immunity against challenge infection. Their reduced virulence and survivability confirms their vaccine candidature and demands further investigations. However, similar attempts with *A2* (amastigote specific expression 2) genes failed due to their multiplicity and rapid compensation by amplification of the remaining genes.

### Proteases

Proteases play key roles in the life cycle, host–parasite relationship and pathogenesis of parasitic diseases. The deletion of genes for cathepsin B cysteine protease, oligopeptidase B serine protease, or subtilisin protease resulted in avirulent strains causing proteome remodeling, upregulation of gene-transcription in macrophages, or reduced promastigote to amastigote differentiation *in vitro*, respectively. As in many other diseases, proteases form attractive drug targets.

### Cytoskeletal elements

Some flagellar components were also found to play important roles in the parasites life cycle. The deletion of *myosin XXI*, that encodes a novel class of myosin; the 70 kDa subunit of the outer dynein arm docking complex; a novel actin related protein (ORF LmjF.13.0950) or the over-expression of *ARL-3A* (ADP-ribosylation factor like protein), a homolog of human ARL-3, all resulted in impairment of flagellar assembly, motility, and survival. They also affected intra-cellular trafficking, virulence *in vitro* and mitochondrial membrane potential to various extents. Hence, a novel group of putatively essential components that hold promise for further studies were identified.

In addition to these, components of some other pathways have also been manipulated to assess their functional role and dispensability. Heterozygous mutants of glyoxalase I (*GLO I*), involved in methylglyoxal metabolism and *CYP5122A1*, involved in xenobiotic metabolism and sterol biosynthesis, impaired growth, mitochondrial membrane potential, and normal metabolism. Altered drug susceptibility and virulence were also observed in the latter mutants. Moreover, attempts at homozygous deletions did not permit survival. In addition, knock-outs of some chaperone proteins like *HSP70-II, HSP90*, and co-chaperones like *SGT* (small glutamine rich tetra trichopeptide) also had deleterious effects. Also, trials of *LiHSP70-II* null mutants to provide protection against *L. major* infection model demonstrated both safety and protection. In another study, the over-expression of a kinase, *CK1.4* (casein kinase 1 isoform 4), increased virulence and metacyclogenesis. As seen, majorly these studies implicate the therapeutic potential of the target genes. Simultaneous evaluation of their LAV potential would greatly fasten the search for an ideal leishmanial vaccine.

## Challenges and Scope for the Future

Although a large proportion of currently licensed vaccines are based on inactivated or whole live attenuated organisms, the scope of LAV gets largely restricted due to safety issues. Foremost, is the risk of reversion to wild type or expression of compensatory genes. The *Leishmania* genome being highly plastic, this has a high probability. Additionally, critical consideration of the position of knock-outs, their effects on upstream and downstream genes, the restriction to manipulate only amastigote stage specific and single copy genes and availability of few selectable markers limits the potential targets and simultaneous multi-gene targeting, respectively (12). Furthermore, the retention of antibiotic resistance genes (20) and generation of cross resistance to anti-leishmanial drugs as in the case of neomycin to paromomycin is undesirable (83). Moreover, prior to human clinical trials, the cultivation of parasites in serum free media, their large-scale production, storage, validation of the best challenge methods-syringe or sandfly mediated, and many months of post challenge follow-up impose practical and as yet unresolved issues (84). In contrast, subunit and DNA vaccines are relatively safe and without these limitations. However, the low predictive power of available pre-clinical models to determine the human outcome of vaccination and the lack of knowledge of convincing markers to monitor their safety or efficacy remain common to all vaccination strategies (2).

The following road map may be considered a basic guideline while working with live attenuated vaccines. Preliminary phenotypic and genotypic screening of the parasites after each recombination event should be followed by vigorous *in vitro* studies on human cell lines. The parasites compartmentalization, proliferation, cellular responses, and activation markers should be closely monitored (85). After successful *in vitro* screening, the *in vivo* experiments in Golden Syrian hamsters and BALB/c mice models should be supported by those on chimeric humanized mice (25, 86). Continuous monitoring assays to test for reversion or attenuation retention by sensitive molecular biology techniques like PCR, microarrays should be done (87). Timely splenic biopsies for parasite load and multiparametric FACS analysis and ELISA for monitoring cytokine responses would help in elucidating the immune correlates of protection or disease development (6). Additionally, the comparison of these results among different groups, namely asymptomatic carriers, non-endemic healthy, endemic healthy, infected-cured, and infected individuals would greatly enhance our knowledge of disease pathogenesis. With the advent of modern imaging techniques, bioluminescent parasites can provide unsurpassable insight at each level of disease progression in real time (beginning from host cell–parasite interaction to dissemination and homing to various organs) also requiring lower number of animals to obtain statistically significant data (88). Lastly, human trials to provide proof of concept studies would strengthen our hypothesis derived from pre-clinical studies.

Parasite gene deletion mutants have helped in numerous pathway studies and elucidation of novel drug targets and vaccine candidates (Table 1). They also offer the possibility of co-administration with adjuvants or drugs to improve disease outcome. Moreover, vectored formulations in recombinant vaccinia (89), *Lactobacillus* (90), adenovirus, or *Salmonella* (91) carriers offer non-pathogenic and genetically modifiable alternatives for safe mucosal delivery, the major entry portal of pathogens. The concept of the flying vaccinator, genetically engineered blood-feeding insects to deliver vaccines to replace mosquito populations is a novel attempt tried in antimalarial programs and can be applied for sandfly eradication (92) too. Lastly, well-defined clinical trials with attenuated parasites will enhance the number of potential therapeutic targets, which are urgently needed to combat leishmaniasis.

## Conflict of Interest Statement

The authors declare that the research was conducted in the absence of any commercial or financial relationships that could be construed as a potential conflict of interest.
